# Strength characterization of knee flexor and extensor muscles in Prader-Willi and obese patients

**DOI:** 10.1186/1471-2474-10-47

**Published:** 2009-05-06

**Authors:** Paolo Capodaglio, Luca Vismara, Francesco Menegoni, Gabriele Baccalaro, Manuela Galli, Graziano Grugni

**Affiliations:** 1 Orthopaedic Rehabilitation Unit and Clinical Lab for Gait Analysis and Posture, Ospedale San Giuseppe, Istituto Auxologico Italiano IRCCS, Piancavallo (Verbania), Italy; 2 Bioengineering Department, Politecnico di Milano, Milano, Italy; 3 Division of Auxology, Ospedale San Giuseppe, Istituto Auxologico Italiano IRCCS, Piancavallo (Verbania), Italy

## Abstract

**Background:**

despite evidence of an obesity-related disability, there is a lack of objective muscle functional data in overweight subjects. Only few studies provide instrumental strength measurements in non-syndromal obesity, whereas no data about Prader-Willi syndrome (PWS) are reported. The aim of our study was to characterize the lower limb muscle function of patients affected by PWS as compared to non-syndromal obesity and normal-weight subjects.

**Methods:**

We enrolled 20 obese (O) females (age: 29.1 ± 6.5 years; BMI: 38.1 ± 3.1), 6 PWS females (age: 27.2 ± 4.9 years; BMI: 45.8 ± 4.4) and 14 healthy normal-weight (H) females (age: 30.1 ± 4.7 years; BMI: 21 ± 1.6). Isokinetic strength during knee flexion and extension in both lower limbs at the fixed angular velocities of 60°/s, 180°/s, 240°/s was measured with a Cybex Norm dynamometer.

**Results:**

the H, O and PWS populations appear to be clearly stratified with regard to muscle strength.: PWS showed the lowest absolute peak torque (PT) for knee flexor and extensor muscles as compared to O (-55%) and H (-47%) (P = 0.00001). O showed significantly higher strength values than H as regard to knee extension only (P = 0.0014). When strength data were normalised by body weight, PWS showed a 50% and a 70% reduction in PT as compared to O and H, respectively. Knee flexors strength values were on average half of those reported for extension in all of the three populations.

**Conclusion:**

the novel aspect of our study is the determination of objective measures of muscle strength in PWS and the comparison with O and H patients. The objective characterization of muscle function performed in this study provides baseline and outcome measures that may quantify specific strength deficits amendable with tailored rehabilitation programs and monitor effectiveness of treatments.

## Background

Obesity has a profound relationship with disability and, at severe levels, is itself disabling in terms of mobility and exercise capacity. Excessive body weight is an independent risk factor for various conditions many of which are disabling (i.e. stroke, diabetes, osteoarthritis, etc). Furthermore, many persons with an existing disability may become obese often due to impaired mobility and activity limitations. As the prevalence of obesity is increasing at an alarming rate worldwide, obesity-related disabilities will eventually become a serious threat to national health systems, specially if, in regard to children and adolescents, they occur in early life and continue for a longer life span.

Despite this evidence, supported by more than 100 papers published in the last two years with the key-words "obesity and disability", the physiopathological determinants of the obesity-related disability have been scarcely investigated.

Prader-Willi syndrome (PWS) is the most frequent cause of syndromic obesity and occurs in 1 in 25,000 live births. Its major clinical features include muscular hypotonia, childhood-onset obesity, short stature, small hands and feet, scoliosis, hypogonadism and developmental delay, resulting from a loss of the paternal copy of chromosome15q11-q13. The syndrome has two phases. Initially, PWS is characterized by severe neonatal hypotonia, feeding problems and a failure to thrive. This is followed by hyperphagia and weight gain between the ages of 1 and 6, leading most PWS subjects to develop morbid obesity. Both hypotonia and obesity affect the development of motor and functional skills that children usually learn at that age [1]. PWS children's ability in sitting, kneeling, standing and walking is delayed compared with children with the same age. These patients develop their typical gait pattern already influenced by obesity. In adult life, the progressive effects of obesity on joints, small feet, hypotonia and the other orthopaedic problems produce further gait deviations [2].

Typically, PWS patients present with reduced lean body mass and increased fat to lean mass ratio not only if compared with lean subjects but also in relation to simple obese patients [3,4]. Body composition abnormalities of PWS subjects resemble that of individuals with severe growth hormone deficiency or even of frail elderly people [5]. In this respect, administration of growth hormone has been shown to increase lean body mass and decreases fat mass, and to improve physical strength, and agility [6]. However, long-term treatments appear to only partially compensate the lean mass deficit [7].

Decreased muscle mass represents the proximate cause of the hypotonia but the pathogenesis of this condition is unclear. Neuromuscular studies and muscular ultrasound analysis are usually normal [8]. Histologic and ultrastructural evaluation of muscle fibers in PWS are scarce. Sone [9] reported histochemical abnormalities such as fiber size variation of both type 1 and 2 fibers, type 2 fiber atrophy, increased number of type 2C fibres and decreased number of type 2B fiber, thus suggesting that primary muscle pathology play a role in hypotonia and weakness. Other studies demonstrated an abnormal proportions of type 2 muscle fiber subtypes[10-12]

In addition to such relative sarcopenia and abnormal muscle fibres composition, PWS patients show a reduced pattern of spontaneous physical activity [13]. Evidence exists that a short (up to 10 minutes) daily muscle training programs in a home setting may increase lean mass and spontaneous physical activity, as assessed by DEXA and pedometer registration, respectively [14]. Adequate increase in muscle mass in response to enhanced physical activity seems to point to the diminished spontaneous physical activity as the main cause for decreased muscle mass in PWS subjects, but further research is needed.

Together with the determination of muscle mass and fibres composition, objective characterization of muscle function may ultimately help identifying specific deficits amendable with tailored rehabilitation programs and providing baseline and outcome measures. From a functional perspective, to date there are only few papers providing quantitative data of lower limb muscle strength in non-syndromal obese subjects [15-17]. Physical strength and functional capacities of PWS subjects has been previously performed by manual non-instrumental testing [13] and with a modified Bruininks-Oseretsky test [6].

The literature fails to provide objective instrumental data regarding physical capacities in PWS. The objective characterization of muscle function could quantify specific strength deficits and provide baseline and outcome measures in the rehabilitation of PWS.

In this wake, the aim of our study was therefore to provide some preliminary reference data of lower limb muscle strength in a group of adult patients with PWS using a dynamometer. In order to ascertain whether differences in performance are related solely to excessive body weight, the results were compared with those obtained both in patients affected by non-syndromal obesity and in a group of normal-weight subjects.

## Methods

### Subjects

Over a period of six months, 26 female subjects consecutively admitted to our rehabilitation institute were enrolled in this study (Additional file 1): 20 obese (O), age mean 29,1 years (range, 20–40); Body Mass Index (BMI) mean 38.1 (range, 31–43), and 6 PWS, age mean 27.2 years (range, 21–36); BMI mean 45.8 (range, 38–51). In addition, a control group of 14 healthy normal-weight (H) age mean 30.1 years (range, 23–38); BMI mean 21 (range 19–23) was recruited. Physical examination included determination of height and weight in fasting conditions and after voiding. BMI was defined as weight/height^2 ^(kg/m^2^). Standing height was determined by a Harpenden Stadiometer, while body weight was measured to the nearest 0.1 kg, by using standardized equipment. PWS patients showed the typical clinical phenotype [18], and all underwent cytogenetic analysis: 4 had interstitial deletion of the proximal long arm of chromosome 15 while 2 had uniparental maternal disomy for chromosome 15. All PWS adults suffered from childhood obesity and were undergoing strict parental guidance for control of feeding obsession. Four PWS females had primary amenorrhea, and the remaining suffered from oligomenorrhea. Characteristic facial features (narrow bifrontal diameter, almond-shaped eyes, and downturned mouth with thin upper lip) as well as small hands and feet were present. Typical behaviour problems were reported, including temper tantrums and obsessive-compulsive features. In addition, all PWS subjects showed a mild mental retardation. In this respect, the inclusion criteria for participating in the study were a score over cut-off value of 24 in the Mini Mental State Examination (MMSE) Italian version [19] in order to obtain a minimum intellective level allowing the administration of the tests. All PWS patients showed normal findings on the main laboratory test, including adrenal and thyroid functions. O group included only patients with essential obesity and BMI > 30. None of them reported orthopaedic conditions limiting the range of motion of the knee. No patient was under any medications expected to alter the results of the study, including growth hormone. Control subjects were all free of pathological conditions and had a sedentary life style.

The study was approved by the Ethic Committee of the Istituto Auxologico Italiano. Written informed consent was given by the patients and, where applicable, the parents.

### Equipment

Knee flexors and extensors strength was assessed using an isokinetic dynamometer (Cybex Norm). Muscle strength was measured in both lower limbs with the subjects in a sitting position with hip flexed at 90°. The trunk and the thigh of lower limb to be tested were tightly secured by velcro-straps to the experimental chair. The mechanical lever arm was aligned to the axis of rotation of the knee. To account for the influence of the gravity effect torque, data were corrected by the weight of the subject's lower limb at 45°. Subjects were tested at three fixed angular velocities: 60°/s, 180°/s, 240°/s. The subjects began each isokinetic contraction with the knee flexed at 90° and continued through the full range of motion.

On the test day, PWS subjects performed several warm-up trials at submaximal levels to ensure a thorough familiarization with the equipment because of their need for routine sameness and consistency in the learning environment. They were then asked to complete five maximal contractions at each angular velocity in a randomized order, but only the best value was retained for further statistical analysis.

All participants were verbally encouraged to perform each test as maximally as possible. Visual feed back during the test was also provided. The same investigator conducted the tests for all the subjects. The impact artefact was not included in the measurement of maximal torque. A recovery of 1 minute was allowed between each maximal contraction, to minimize fatigue.

### Selected parameters

The following parameters were obtained from the maximum isokinetic tests and subsequently analyzed: peak torque (PT, [Nm]), peak torque relative to body weight (PT%BW), angle at peak torque (AnPT, [deg]), and flexor/extensor ratio.

### Statistical Analysis

Unless otherwise noted, all data are presented as mean (one standard deviation, SD). Before using parametric statistical procedures, the assumptions of normality, homogeneity and sphericity were verified. Three-way mixed Analysis of Variance (ANOVA, 2 × 3 × 3 design) was used on each dependent variable (PT, PT%BW, AnPT, and hamstring/quadriceps ratio, measured on the right and left side of flexors and extensors). The independent variables included one within-subject factor, side, with two levels (right and left), one within-subject factor, speed, with three levels (60, 180, and 240°/s), and one between-subject factor, group, with three levels (normal, obese, and Prader-Willi syndrome patients). AnPT violated the normality in 20% of the subgroup data. Visual inspection of the normality plots showed slight deviation from normality. Therefore, we applied both parametric and non-parametric ANOVA (after rank transformation of the data). Since results were similar, to facilitate the reader, we decided to present the results of the parametric analysis to avoid reporting transformed data instead of raw values. Post-hoc analysis was performed using LSD test. Given the descriptive nature of the studies, we presented when possible the raw data and not only the estimated marginal means according to the results of the ANOVAs. The probability of Type I error was set a priori at 0.05 for all statistical analysis.

## Results

### Peak Torque

A significant speed × group interaction was found for extensor PT (F_4,74 _= 12.877, p < 0.001). Estimated marginal means are presented in Additional file 2. Post hoc analysis showed that PT was lower in PWS compared to both O and H (p < 0.001) at all speeds, and in H compared to O (p < 0.001). PT decreased increasing the speed. The PT at 60°/s was higher than 180°/s and 240°/s (p < 0.001) for all groups. Similarly PT at 180°/s was higher than 240°/s for H and O (p < 0.001) and PWS (p = 0.013). No other significant interactions were found (p > 0.769).

A significant speed × group interaction was also found for flexor PT (F_4,74 _= 10.926, p < 0.001). Estimated marginal means are presented in Additional file 2. Post hoc analysis showed that PT was lower in PWS compared to both O and H (p < 0.001) at all speeds, while no differences were found between O and H (p > 0.13).

PT decreased increasing the speed. The PT at 60°/s was higher than 180°/s (p < 0.001) for O and H, and PWS (p = 0.005). Similarly PT at 180°/s was higher than 240°/s for H and O (p < 0.001) and PWS (p = 0.009). No other significant interactions were found (p > 0.527).

### Peak torques relative to body weight

Similar results were obtained for PT%BW. A significant speed × group interaction was found for extensor PT%BW (F_4,74 _= 29.686, p < 0.001). Estimated marginal means are presented in Additional file 3. Post hoc analysis showed that PT%BW was lower in PWS compared to both O and H (p < 0.001) at all speeds, and in O compared to H (p < 0.001). PT%BW decreased increasing the speed: the PT%BW 240°/s was lower than 180°/s (p < 0.001) for all groups. Similarly PT%BW 180°/s was lower than 60°/s for H and O (p < 0.001), and PWS (p = 0.003). No other significant interactions were found (p > 0.813).

Even for flexors a significant speed × group interaction was found (F_4,74 _= 48.052, p < 0.001). Estimated marginal means are presented in Additional file 3. Results were similar to those pertaining the extensors, with post hoc analysis showing lower PT%BW in PWS compared to both O and H (p < 0.001) at all speeds, and in O compared to H (p < 0.001). PT%BW decreased increasing the speed: the PT%BW 240°/s was lower than 180°/s for O and H (p < 0.001), and PWS (p = 0.01), as well as 180°/s was lower than 60°/s for H and O (p < 0.001), and PWS (p = 0.004). No other significant interactions were found (p > 0.388).

### Angle at peak torque

A significant speed × group interaction was found for AnPT of extensors (F_4,74 _= 5.683, p < 0.001). Post hoc analysis showed a significant higher value of AnPT between PWS and control groups (PWS vs O: p = 0.004; PWS vs H: p = 0.002) only at maximum speed (Additional file 4). AnPT was influenced by speed in O (p < 0.001) and H (p = 0.003), but not in PWS (p > 0.303). No other significant interactions were found (p > 0.343).

Similarly to extensors, a significant speed × group interaction was found in flexors (F_4,74 _= 9.335, p < 0.001). Post hoc analysis showed significant different values among groups only at 60°/s, with O showing higher AnPT than H (p = 0.008) and PWS showing higher angle than O (p = 0.007) and H (p < 0.001) (Additional file 4). AnPT increased with increasing speed in H among all speeds (p < 0.001) and in O between 180°/s and 240°/s (p < 0.001), while in PWS the angle at 60°/s was higher than 180°/s (p = 0.007). No other interactions were found (p > 0.338)

### PT flexors/extensors ratio

**Figure 1:**
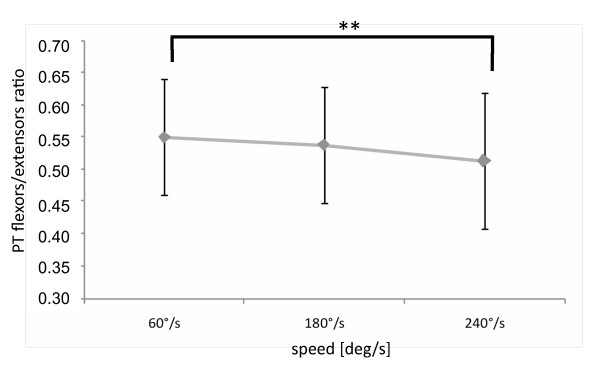
**Speed main effect on flexors/extensors ratio: estimated marginal means and post-hoc analysis**. ** p < 0.01.

**Figure 2:**
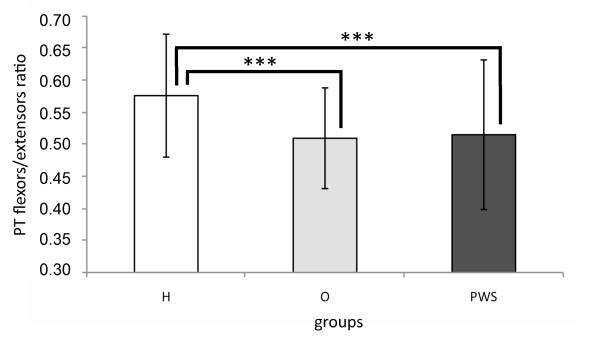
**Group main effect on flexors/extensors ratio: estimated marginal means and post-hoc analysis**. *** p < 0.001.

A significant main effect was found for speed (F_2,74 _= 7.967, p < 0.001) and group (F_2,37 _= 3.650, p = 0.0357). No other significant interactions were found (p > 0.232). Raw data are presented in Additional file 5, while estimated marginal means and post hoc results are presented in Figure 1 and 2.

## Discussion and conclusion

Our results provide an objective functional picture characterizing the muscle function of PWS and obese population as compared to normal-weight control subjects. The novel aspect of our study is the determination of objective measures of lower limbs muscle strength in PWS and the comparison with non-syndromal obese patients.

Efficient lower limb muscles are the key to independent mobility and strength of the flexor and extensor muscles is highly correlated with the capacity to execute daily tasks safely [20]. The relationship between strength and mobility tasks is curvilinear, suggesting that other factors than muscle function contribute to mobility, still muscle strength is considered the most important factor to enhance mobility [21,22]. It has been shown that reduced muscle strength relative to body weight induces earlier fatigue of the quadriceps muscles in the obese which, in turn, reduces shock attenuation and increases the loading rate and variability at the knee during gait [23]. Quadriceps muscle strength is known to be an important factor governing the vibration dampening of the lower limb [24] and its weakness is known to be a risk factor for the development of knee osteoarthritis [25-30]. Lower rates of musculoskeletal loading have been found in strength-trained females when compared with sedentary females walking at similar speeds, suggesting that increased muscular strength may enhance shock attenuation [31]. Despite the implications of such biomechanical data, there is scanty evidence of functional quantification in overweight patients. Physical strength in PWS has been previously analyzed only by means of non-instrumental functional tests [6]. In our study, the H, O and PWS populations appear to be clearly stratified with regard to muscle strength, and PWS showed the lowest absolute peak torque for knee flexor and extensor muscles at all three angular velocities as compared to H (-47%) and O (-55%) (Tab. 2). Our data show that PWS were the least performing subjects. Reduced muscle tone, early childhood obesity, an impaired muscular trophic response could negatively interfere with muscular strength and coordination development, as well as with motor planning skills and account for the lowest absolute strength measured. On the other hand, it is not possible to exclude that the impairment of lower limb muscle function associated with PWS may be secondary to different mechanisms from those observed in patients with non-syndromic obesity, e.g. primary muscle pathology. The comparison of syndromal and non-syndromal obese subjects suggests that other factors than obesity *per se *seem to contribute to reduced muscular strength in PWS. However, we did not measure physical activity levels in PWS since surveys and recall instruments are scarcely reliable in a population that has difficulty recalling such information [32].

Our PWS had a significantly higher BMI as compared to non-syndromal obese patients (p < 0.01). When PWS strength data were normalised for BW a 70% and a 50% reduction in PT as compared to H and O, respectively, was evident. The percentage strength loss of PWS as compared to O appears unaffected by normalization, suggesting that other factors than obesity contribute to reduce muscular strength in PWS, while the difference between PWS and H increased after taking BW into account.

Non-syndromal obese subjects produced greater absolute isokinetic torque than their lean counterpart, which is in line with recent reports [33,34]. Absolute strength is higher in O than H possibly due to a higher absolute fat-free mass rate in O, which is correlated to BMI. It could be therefore hypothesised that obese patients retain a greater muscle mass in response to a greater loading, which could act as training stimulus. However, when data are normalized per BW, O showed significantly lower knee flexors and extensors strength as compared to H.

Normalization per BW instead of the more common normalization per fat-free mass was chosen to represent in some way the load bearing on the muscles, which is in actual fact one of the major biomechanical constraint in obese subjects. In our opinion, normalization per BW may provide a realistic picture of the functional capacity of obese patients with regard to their BW. In other terms, such normalization may appropriately reflect the disabling degree of the motor limitation of obese subjects and represent a more meaningful indicator of physical capacity. In fact, it has been shown that muscle power, hence strength under dynamic conditions, normalized per BW is reliably related with functional performance in obese patients [35].

Duvigneaud [15] and Maffiuletti [16] reported that muscle strength per unit of fat-free mass was similar in obese and non-obese adolescents, suggesting that obesity has little or no effect on quadriceps muscle function characteristics. Expectedly, by normalizing per fat-free mass, which is an indirect indicator of muscular mass, strength differences between obese and controls disappear. Another reason for discrepancy with our data may be related to the different age of the patients. Interestingly, the same authors [15,16] speculated that the reduced functional capacity of obese adolescents during motor tasks involving squatting and kneeling could be secondary to the obese producing their maximal strength at shorter muscle length (40° knee flexion) rather than at 80° knee flexion, where their lean counterparts obtained the peak values [16]. However, our data on the angle at which peak strength was achieved are in contrast with those previous observations, with PT angles non significantly different among PWS, obese patients and controls, except for the knee extensors at higher angular speed (240°/s) and knee flexors at lower speed (60°/s), where PWS seem to obtain peak strength values at greater knee angles than O and H.

We decided to measure strength under three conditions of angular velocities (low: 60°/s, moderate: 180°/s, high: 240°/s) to provide a comprehensive profile of individual muscular strength and power [36].

The significant speed × group interaction we found in PT is probably the result of the lower absolute extensor decline in the PWS group (-32.4 Nm) than H (-52.4 Nm) and O (-59.8 Nm). A similar decline was observed in flexors' PT: -19.4 Nm in PWS group, -33.2 Nm in H and -33.9 Nm in O. When normalizing per body weight the same behavior was evident. Even more, we observed a more evident strength gap at higher angular velocities in both knee flexion and extension.

This seems to suggest that muscle power more than strength might be reduced primarily in PWS and in obese as compared to normal-weight subjects. Muscle power is highly correlated with mobility tasks, describing more of the variance than does isometric strength in these tasks. Short-term performance, such as raising from a chair, however, showed higher correlations with muscle strength rather than power [22]. This is probably due to the fact that strength *per se *rather than the rate at which force is generated is important in such tasks. However, muscular power output depends from a combination of factors including muscle strength and fibres composition and neural drive. In addition to strength data, bioptic evidence on fibers composition and functional investigation on motor units recruitment in PWS and obese patients will be needed to unveil the physiological basis of our results.

In line with the above mentioned studies [15,16], we observed that the knee flexion strength values were on average half of those reported for extension in the O and H groups, with significant differences for all reported parameters (P < 0.01). The same results were obtained in PWS subjects. The ratio between flexor and extensor muscle strength appears to be altered in the O and PWS populations as compared to H. Specifically, the lower ratio in the O group is due solely to higher extensor strength as compared to H. This could be explained by a training effect on the anti-gravitary muscles (i.e.: extensors) caused by the exposure to higher body weight. The same effect could account for the ratio value in the PWS population, although their muscle strength values were approximately half of those observed in O.

It has been previously reported that the stimulation of motor activity, through its positive action on lean mass, muscular strength and energy balance, may contribute to increase the physical performances of patients with PWS [13,14]. In this wake, our study provides some initial reference values for further research on muscle function of adult patients with PWS. A possible bias of our study is the low sample size which decreases its external validity. It should however be beared in mind that PWS is a rare syndrome and muscle strength is gender-specific. We decided to limit our investigation to female subjects and therefore only a small number of PWS patients could be recruited.

In general, few data are reported in the recent literature on the obesity-disability relationship, even if obesity should be acknowledged as a cause of disability. BMI is certainly related to the individual functional capabilities [37,38], but should not be considered the only measurement of obesity-related physical impairment. In this context, there is a need for functional measurements that can be more predictive of the actual individual physical capacities. The objective characterization of muscle function performed in this study provides baseline and outcome measures that may quantify specific strength deficits amendable with tailored rehabilitation programs and monitor effectiveness of treatments.

## Competing interests

The authors declare that they have no competing interests.

## Authors' contributions

PC conceived the study, participated in its design and coordination, drafted the manuscript. LV carried out the strength measurements and collected the data. FM participated in the design of the study, performed the statistical analysis, drafted the manuscript. GB helped carrying out the strength measurements and supervised the measurements. MG performed the statistical analysis. GG recruited the patients, participated in the design of the study, drafted the manuscript. All authors read and approved the final manuscript.

## Pre-publication history

The pre-publication history for this paper can be accessed here:



## Supplementary Material

Additional file 1 **Table 1. **Age and body mass index of the considered subjects. The data provided represent the mean values for age and body mass index of the considered subjects. Click here for file

Additional file 2 **Table 2 – Peak torque (PT) values expressed as Nm**. Estimated marginal mean values of peak torque are presented for the three experimental groups. Click here for file

Additional file 3 **Table 3 – Mean Peak Torque values expressed in percent of body weight (PT%BW)**. Estimated marginal mean values of peak torque expressed in percent of body weight are presented for the three experimental groups. Click here for file

Additional file 4 **Table 4 – Angle (°) at Peak Torque (AnPT) values**. Mean values of angles at Peak Torque are presented for the three experimental groups Click here for file

Additional file 5 **Table 5 – Descriptive flexors/extensors ratio data**. Mean values of the flexors/extensors ratio are presented for the three experimental groups. Click here for file
