# Supplementary material for: Strength characterization of knee flexor and extensor muscles in Prader-Willi and obese patients
Source: BMC Musculoskelet Disord. 2009 May 6;10:47. doi: 10.1186/1471-2474-10-47 (PMC2685367; doi:10.1186/1471-2474-10-47)
Supplement: Additional file 1 — Table 1. Age and body mass index of the considered subjects. The data provided represent the mean values for age and body mass index of the considered subjects. [file 1471-2474-10-47-S1.doc]

***Table 1.***

|  |  | H | O | PWS |
| --- | --- | --- | --- | --- |
| Age [years] |  | 30.1 (4.7) | 29.1 (6.5) | 27.2 (4.9) |
| BMI [kg/m^2^] | *,**,+ | 21.0 (1.6) | 38.1 (3.1) | 45.8 (4.4) |

*Table 1. Age and body mass index of considered subjects.*

** statistically significant difference between PWS-H (P<0.01)*

*** statistically significant difference between PWS-O (P<0.01)*

*+ statistically significant difference between H and O (P<0.01).*
