# Supplementary material for: Strength characterization of knee flexor and extensor muscles in Prader-Willi and obese patients
Source: BMC Musculoskelet Disord. 2009 May 6;10:47. doi: 10.1186/1471-2474-10-47 (PMC2685367; doi:10.1186/1471-2474-10-47)
Supplement: Additional file 2 — Table 2 – Peak torque (PT) values expressed as Nm. Estimated marginal mean values of peak torque are presented for the three experimental groups. [file 1471-2474-10-47-S2.doc]

|  | H (n=14) | O (n=20) | PWS (n=6) | Post hoc |
| --- | --- | --- | --- | --- |
| *Extensors* |  |  |  |  |
| 60°/s | 123.8(16.1) | 146.6(21.1) | 68.7(16.3) | O>H>PWS *** |
| 180°/s | 87.0(10.0) | 104.7(18.0) | 47.4(10.5) | O>H>PWS *** |
| 240°/s | 71.4(9.1) | 86.8(15.5) | 36.3(9.5) | O>H>PWS *** |
| Post hoc | 60>180>240 °/s  *** | 60>180>240 °/s  *** | 60>180>240 °/s * |  |
| *Flexors* |  |  |  |  |
| 60°/s | 73.4(11.4) | 75.9(8.7) | 37.2(8.1) | O = H >PWS *** |
| 180°/s | 50.0(8.1) | 54.1(7.2) | 24.7(7.9) | O = H >PWS *** |
| 240°/s | 40.2(8.3) | 42.0 (6.5) | 17.8(6.0) | O = H >PWS *** |
| Post hoc | 60>180>240 °/s  *** | 60>180>240 °/s  *** | 60>180>240 °/s  ** |  |

Table 2 - Peak torque (PT) values expressed as Nm.

Data are reported as mean (SD), *** p<0.001, ** p<0.01, * p<0.05
