# Supplementary material for: Strength characterization of knee flexor and extensor muscles in Prader-Willi and obese patients
Source: BMC Musculoskelet Disord. 2009 May 6;10:47. doi: 10.1186/1471-2474-10-47 (PMC2685367; doi:10.1186/1471-2474-10-47)
Supplement: Additional file 3 — Table 3 – Mean Peak Torque values expressed in percent of body weight (PT%BW). Estimated marginal mean values of peak torque expressed in percent of body weight are presented for the three experimental groups. [file 1471-2474-10-47-S3.doc]

|  | H (n=14) | O (n=20) | PWS (n=6) | Post hoc |
| --- | --- | --- | --- | --- |
| *Extensors* |  |  |  |  |
| 60°/s | 216(25.1) | 147.7(21.3) | 71.5(20.3) | H>O>PWS *** |
| 180°/s | 151.8(12.9) | 105.2(16.7) | 49.1(12.4) | H>O>PWS *** |
| 240°/s | 124.4(11.1) | 87.1(14.5) | 37.8(11.8) | H>O>PWS *** |
| Post hoc | 60>180>240 °/s  *** | 60>180>240 °/s  *** | 60>180>240 °/s  ** |  |
| *Flexors* |  |  |  |  |
| 60°/s | 128.0(16.0) | 76.1(8.4) | 38.4(9.7) | H>O>PWS *** |
| 180°/s | 87.0(12.6) | 54.1(6.6) | 25.1(8.4) | H>O>PWS *** |
| 240°/s | 69.8(13.0) | 42.0(6.6) | 18.3(6.9) | H>O>PWS *** |
| Post hoc | 60>180>240 °/s  *** | 60>180>240 °/s  *** | 60>180>240 °/s  ** |  |

Table 3 – Peak Torque values expressed in percent of body weight (PT%BW). Data are reported as mean (SD), *** p<0.001, ** p<0.01.
