# Supplementary material for: Strength characterization of knee flexor and extensor muscles in Prader-Willi and obese patients
Source: BMC Musculoskelet Disord. 2009 May 6;10:47. doi: 10.1186/1471-2474-10-47 (PMC2685367; doi:10.1186/1471-2474-10-47)
Supplement: Additional file 4 — Table 4 – Angle (°) at Peak Torque (AnPT) values. Mean values of angles at Peak Torque are presented for the three experimental groups [file 1471-2474-10-47-S4.doc]

|  | H (n=14) | O (n=20) | PWS (n=6) | Post hoc |
| --- | --- | --- | --- | --- |
| *Extensors* |  |  |  |  |
| 60°/s | 59.6(2.3) | 61.5(5.1) | 58.1(8.4) |  |
| 180°/s | 54.3(4.1) | 56.4(3.0) | 55.8(5.4) |  |
| 240°/s | 51.8(3.4) | 52.4(2.1) | 57.2(6.0) | H=O<PWS ** |
| Post hoc | 60>180>240 °/s ** | 60>180>240 °/s *** |  |  |
| *Flexors* |  |  |  |  |
| 60°/s | 23.3 (4.3) | 31.6 (11.2) | 42.9 (4.2) | H<O<PWS ** |
| 180°/s | 31.5 (3.6) | 33.1 (5.0) | 32.8 (3.9) |  |
| 240°/s | 38.5 (4.0) | 39.0 (3.5) | 36.3 (10.0) |  |
| Post hoc | 60<180<240 °/s *** | 60=180<240 °/s ** | 60>180=240 °/s ** |  |

Table 4 - Angle (°) at Peak Torque (AnPT) values.

Data are reported as mean (SD). *** p<0.001, ** p<0.01.
