# Supplementary material for: Strength characterization of knee flexor and extensor muscles in Prader-Willi and obese patients
Source: BMC Musculoskelet Disord. 2009 May 6;10:47. doi: 10.1186/1471-2474-10-47 (PMC2685367; doi:10.1186/1471-2474-10-47)
Supplement: Additional file 5 — Table 5 – Descriptive flexors/extensors ratio data. Mean values of the flexors/extensors ratio are presented for the three experimental groups. [file 1471-2474-10-47-S5.doc]

|  | H (n=14) | O (n=20) | PWS (n=6) |
| --- | --- | --- | --- |
| *PT Flexors/extensors ratio* | |  |  |
| 60°/s | 0.593 (0.071) | 0.520 (0.054) | 0.550 (0.118) |
| 180°/s | 0.574 (0.087) | 0.520 (0.066) | 0.514 (0.106) |
| 240°/s | 0.562 (0.103) | 0.489 (0.081) | 0.480 (0.095) |

Table 5. Descriptive flexors/extensors ratio data. Data are reported as mean (SD).
